# Overcoming Immune Evasion in *Staphylococcus aureus*: Strategies for Rational Vaccine Design

**DOI:** 10.1021/acsinfecdis.5c00569

**Published:** 2025-09-12

**Authors:** Khyber Shinwari, Brenda Vieira, Isabelle Ciaparin, Anders P. Hakansson, Michelle Darrieux, Thiago Rojas Converso

**Affiliations:** † Laboratório de Biologia Molecular de Microrganismos, 154623 Universidade São Francisco, Bragança Paulista 12916-900, Brazil; ‡ Division of Experimental Infection Medicine, Department of Translational Medicine, 5193 Lund University, Lund 223 62, Sweden

**Keywords:** Staphylococcus aureus, reverse vaccinology, immune evasion, omics technologies, vaccine design

## Abstract

*Staphylococcus aureus* remains one of the most elusive targets in bacterial vaccinology, primarily due to its complex immune evasion strategies and the phenomenon of immune imprinting. Despite decades of research and numerous clinical trials, no vaccine has demonstrated protective efficacy in humans. This review examines the underlying reasons for these failures and proposes a rational, integrative framework for next-generation vaccine development. Recent advances in reverse vaccinology, omics-driven antigen discovery, immunoinformatics, and artificial intelligence are highlighted as tools to identify conserved, immunogenic, and subdominant antigens. The review also discusses approaches for neutralizing virulence factors, disrupting biofilm-associated mechanisms, and circumventing dysfunctional immune memory. Particular emphasis is placed on the design of multivalent vaccine formulations capable of addressing the antigenic redundancy and immune modulation employed by *S. aureus*. By aligning systems biology with precision vaccinology, this review outlines a translational strategy to overcome the long-standing obstacles in the development of a safe and effective *S. aureus* vaccine.

## Graphical abstract

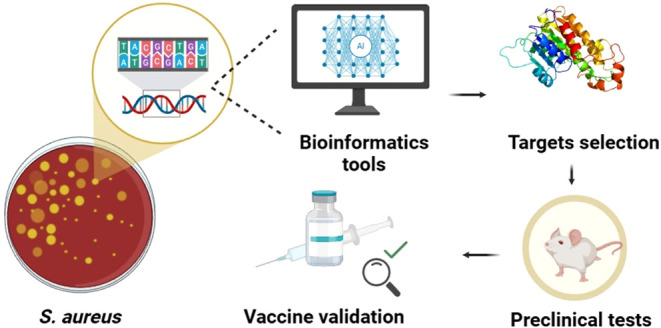

## 1 The Enduring Challenge of *Staphylococcus aureus* Vaccine Development

### 1.1 Clinical Burden and Global Impact

*Staphylococcus aureus* represents a widespread bacterial pathogen associated with infections ranging in severity from localized skin and soft tissue infections (SSTIs) to severe life-threatening diseases such as pneumonia, sepsis, osteomyelitis, and endocarditis.^1^ The clinical burden and global impact of *S. aureus*, particularly its methicillin-resistant strains (MRSA), present significant challenges to public health. The prevalence of *S. aureus* infections is substantial, with global rates estimated at 24.8%, and MRSA specifically affecting 5.8% of cases.^2^ It is global impact results in significant morbidity, and during bacteremia the case-fatality rate approaches 18% in developed nations and rises further in developing countries, thereby establishing *S. aureus* as an important global health challenge.^1^ Worse still, there are multiple emerging strains of the bacterium which are multidrug resistant, including MRSA, making it increasingly difficult to treat the infection, using existing antibiotics.^3^ A meta-analysis found a 14.69% prevalence of MRSA among residents in elderly care centers.^4^ The economic implications are profound, as MRSA infections lead to increased hospital costs, longer stays, and higher mortality rates.^5^ In China, MRSA infections resulted in hospital costs ranging from 3,220 to 9,606 with extended hospital stays of 6 to 14 days.^5^

### 1.2 Historical Landscape of Vaccine Failures: Lessons from Clinical Trials

The history of vaccine failures for *S. aureus* has been long and full of unsuccessful attempts over the course of a great more than a century, with many well documented failures and a few trials that were not published. Due to the impact of *S. aureus* upon human health, the development of an effective vaccine remains an urgent public health priority. Nevertheless, this effort has been hindered by complex immunological barriers and the pathogen’s remarkable adaptability to host defenses. The apparent failure of vaccine development can be explained by several factors: (i) a focus on humoral immunity related to the potential development of antibodies, (ii) the complexities of the pathogen itself, (iii) the unknown and poorly characterized protective biomarkers, and (iv) lack of good animal infection models. The attempt to develop a vaccine against *S. aureus* dates back to 1902, and there have been continued attempts to develop a vaccine against *S. aureus*, to date there has not been a vaccine that has been shown to be beneficial.^6^ Early trials focused on single-target vaccines, which often failed to elicit robust immune responses.^7^ A critical barrier has been the lack of understanding of protective immunity, particularly the role of cell-mediated immunity, which is essential for combating *S. aureus* infections.^8^ Previous vaccines primarily targeted humoral immunity, neglecting the importance of T-helper 17 cells and interleukin 17 in providing protection.^8,9^

Despite more than three decades and 30 randomized clinical trials, no *S. aureus* vaccine has been licensed.^10^ These repeated failures underscore important challenges in understanding and eliciting human immunity to this pathogen.

Some examples of failures include (Table 1):

- **Merck’s Vaccine Candidate (2011):** The Phase III trial of the Merck V710 vaccine was terminated early after enrolling approximately 8000 patients due to lack of efficacy. V710 was a nonadjuvanted subunit vaccine based on the iron-regulated surface determinant B (IsdB), a bacterial surface protein-based antigen.^11–13^ Although V710 elicited significant seroconversion among surgical patients, it failed to prevent postoperative *S. aureus* bacteremia or deep sternal wound infections. Notably, an alarming finding was that vaccinated individuals who subsequently developed postoperative *S. aureus* infections not limited to bacteremia experienced higher mortality rates compared to those receiving placebo. This increased risk of death was hypothesized to result from a dysregulated inflammatory response, potentially reflecting a Th2-skewed immunity rather than the protective Th1 and Th17 responses required for effective clearance of *S. aureus*. These outcomes underscore a substantial gap in our understanding of the interaction between *S. aureus*, the IsdB antigen, and the host immune response, highlighting the complex challenges inherent in developing efficacious vaccines against this pathogen.^11–13^

- **Pfizer’s Vaccine Candidate (2012–2018):** The SA4Ag vaccine, composed of capsular polysaccharide conjugate types 5 and 8, a mutant ClfA protein, and the MntC subunit, targets key virulence factors of *S. aureus*. In preclinical models (rat and mouse), SA4Ag demonstrated a promising level of efficacy, significantly reducing bacterial burden in various invasive infection models and achieving near-complete clearance in an endocarditis model. Although vaccination elicited robust antibody responses in human subjects, the candidate was discontinued during phase 2b clinical trials. Despite inducing immune responses, the incidence of infection in vaccinated patients did not differ significantly from the placebo group. This outcome underscores the persistent challenge of bridging the gap between encouraging animal model data and actual clinical efficacy in humans.^11,14^

- **Monoclonal Antibodies:** Passive immunization approaches employing Arsanis’ ASN100, Aridis’ Suvratoxumab, and Tosatoxumab were also failed to prevent or treat *S. aureus* infections in midphase and Phase 3 clinical trials.^11,15^ A common feature among these failures was the inability to translate protective efficacy observed in preclinical models, especially murine models, into meaningful protection in humans.^1^ This gap demonstrates a major disjunction in immune responses or pathogen relationships across these models and humans.^11^

**Table 1:** Key Failures in *S. aureus* Vaccine Development

**vaccine platform**	**target antigens**	**clinical phase**	**trial outcome**	**scientific insights**	**key references**
Merck’s (V710)	IsdB	phase 3	halted due to lack of efficacy; increased mortality in vaccinated infected patients.	amplified inflammatory response; fundamental misunderstanding of *S. aureus* immune interaction.	^9,12^
Pfizer’s (SA4Ag)	four antigens	phase 2b	discontinued; no reduction in infection rate despite antibody generation.	mouse model limitations; successful preclinical data did not translate to humans.	^9,14^
monoclonal antibodies (ASN100, Suvratoxumab, Tosatoxumab)	various	midphase to phase 3	failed to prevent/treat infections.	*S. aureus* manipulates immune system, rendering classic strategies ineffective; nonprotective endogenous antibodies.	^9,15^
general observation	various	all phases	consistent failure despite preclinical success.	immune imprinting; *S. aureus* evades immune system from initial encounter; nonprotective antibodies preferentially recalled.	^1^

Methods employed in failed or terminated *S. aureus* vaccine trials are Polysaccharide-based vaccines use StaphVAX - bivalent capsular polysaccharides (CP5, CP8) conjugated to Pseudomonas exotoxin A - failed in hemodialysis patients due to waning immunity and no protection despite antibody titers that were high.^16^

V710 was a single surface protein vaccine that targeted IsdB (iron-regulated surface determinant B). Increased mortality was noted in infected patients following cardiac surgery; we speculate that a skewed immune response could be detrimental, and Multicomponent protein vaccines (SA4Ag) combine ClfA, MntC, CP5, and CP8. While immunogenic, none demonstrated efficacy and protection against surgical site infections.^17^ Toxoid-based vaccines like PVL toxoid or Hla toxoid monotherapy have solid neutralizing titers, but limited protection in humans may reflect the redundant functional virulence factors.^18^

### 1.3 Complex Host–Pathogen Interactions and Immune Evasion Mechanisms

*S. aureus* has an unusual and long-lasting association with humans, often as a commensal organism - colonizing up to 30% of people without causing disease then eventually becoming lethal.^19^ This close relationship provided *S. aureus* with ample opportunity to develop an array of sophisticated immune evasion strategies that pose significant challenges to vaccine development and therapeutic interventions.^20^ The pathogen can mimic host molecules to evade immune detection, disrupt immune signaling pathways through secreted enzymes, and inhibit key components of immune system such as pattern recognition receptors and the complement cascade. Additionally, Cationic Antimicrobial Peptides (CAMPs) which play a key role in skin protection are targets for mechanisms of resistance developed by *S. aureus*. Overall, *S. aureus* can evolve its genetic material rapidly to occupy various environments and adapt to evade host immune responses.^21^

Immune imprinting, also known as original antigenic sin, is a critical factor contributing to the suboptimal efficacy of *S. aureus* vaccines. Early life exposure to *S. aureus* can induce long-lasting immunological memory that biases future immune responses. Upon vaccination, instead of generating a de novo response to the vaccine antigens, the host immune system often preferentially recalls and amplifies these pre-existing memory responses which are frequently non-neutralizing or nonprotective thereby impairing vaccine effectiveness. *S. aureus* appears to exploit this phenomenon by promoting the generation of ineffective antibodies during initial colonization or infection. This imprinted, nonprotective memory interferes with vaccination by directing the immune response toward ineffective pathways instead of new, protective ones.^10,22^

Recent research has begun to uncover the mechanistic basis of this phenomenon. For instance, nonprotective antibodies generated against antigens such as IsdB have been found to exhibit increased α2,3 sialylation a glycosylation modification that reduces opsonophagocytic activity by neutrophils, which are key to bacterial clearance. This alteration in glycosylation results in diminished ability to promote opsonophagocytic killing by neutrophils, which is one of the most important mechanism of bacterial clearance. In addition, *S. aureus* produces Protein A (SpA) that acts as a superantigen that binds to the variable region of human VH3-encoded antibodies.^10,23^ This interaction, either B cell apoptosis or a skewing of the anti *S. aureus* antibody repertoire, may reduce vaccine effectiveness.^10,23^

The identification of such immune-modulating mechanisms offers insight into how *S. aureus* undermines vaccine-induced protection. These findings highlight that merely presenting an antigen is insufficient; vaccines must be designed to overcome the imprinted immune bias and elicit qualitatively distinct, protective responses. Emerging strategies include targeting subdominant antigens that are less likely to recall nonprotective memory, and employing adjuvants that modulate Fc glycosylation to restore antibody effector functions. Such approaches may hold promise in breaking the cycle of immune imprinting and enhancing vaccine efficacy.^10,22^

In addition to immune imprinting, *S. aureus* has a tremendous number of virulence factors that actively disabled critical components of the host immune system.^21^ These include hemolysins, cytolytic toxins, superantigens that subvert T-helper cell responses, surface adhesion proteins, and biofilm-associated mechanisms that facilitate immune evasion and persistence.^2,24^ Many of these factors specifically target immune cells particularly neutrophils, which play a central role in bacterial clearance while also interfering with complement activation, phagocytosis, and both B- and T-cell-mediated adaptive responses.^14,25,26^

Further complicating vaccine development is the remarkable metabolic plasticity of *S. aureus* and its ability to undergo transient genetic and transcriptional adaptations *in vivo*.^25,27^ These dynamic and context-dependent changes allow the bacterium to fine-tune the expression of virulence factors according to environmental cues, contributing to its survival under immune pressure. Moreover, the sheer number of virulence determinants, their functional redundancy, and temporal regulation help explain the repeated failure of single-antigen vaccine candidates.

## 2 Pioneering Strategies for Antigen Discovery and Vaccine Design

### 2.1 Omics-Driven and Computational Approaches

Traditionally, vaccine discovery has been largely empirical and sometimes slow-moving,^28^ but Omics technologies have the ability to provide global perspectives of the molecular determinants of life and their interactions, and offers an unbiased approach to physiological and pathological processes. With the exception of metabolomics, these methods are based on genomics, with genomics being the field that was effectively born with the sequencing of the complete genome of the bacterium *Haemophilus influenzae* in 1995,^29^ and the successful deciphering of the human genome in 2001.^30,31^ Omics studies provide rich detail and breadth of information regarding the information content in DNA (genomics), its temporal transcription into RNA that is transcriptomics, its subsequent translation into proteins (proteomics) and metabolites (metabolomics), and immunoproteomics gives an overview of the immunogenic peptides or proteins produced. The comprehensive perspective afforded by these approaches complements traditional, reductionist strategies that focus on single molecules or pathway. Historically, many attempts to translate interesting and/or promising preclinical data to a successful vaccine for patients have yielded disappointing results. Omics-based methods offer an opportunity to bridge this gap by enhancing our understanding of host–pathogen interactions and the mechanisms underlying protective immunity.

The omics revolution, with its new bioinformatics, expands the possibilities in vaccine research and offers more than just empirical options. The hope is that it can accelerate the process of vaccine development. It allows for what some are calling reverse vaccinology - an unbiased, genomic-based discovery process for candidate vaccine antigens (Figure 1).^32,33^ Reverse vaccinology involves starting with an analysis of the microbial genome, and the open reading frames (ORFs) within, to discover the putative proteome rather than starting with whole organisms, live attenuated or inactivated, or relying solely on prior knowledge of host–pathogen interactions. This genomic analysis can be refined in various ways to narrow the list of candidate antigens to test in preclinical models: (1) A computational comparison will yield information on the level of conservation of proteins both within and between microbial species; (2) software predicting the subcellular localization may be able to filter out molecules accessible to the antibody, namely, proteins that are released by the microorganism or associated with the surface; (3) algorithms to predict T-cell and B-cell epitopes and hence, immunogenicity.^30,33^ Once candidate antigens prioritized *in silico*, they are expressed recombinantly and assessed for immunogenicity and protective potential in preclinical models (Figure 1).^34^

**Figure 1:**
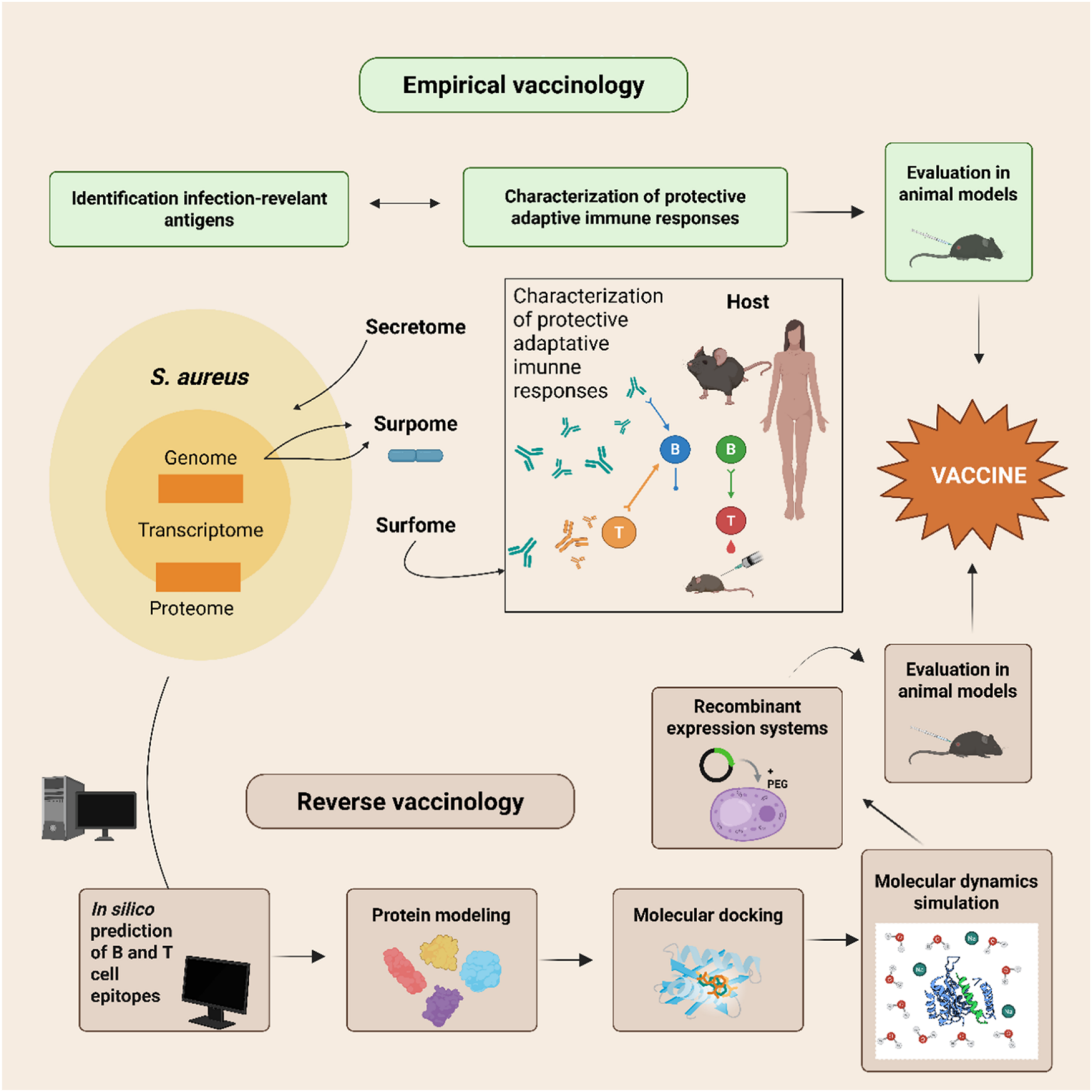
Integrated empirical and reverse-vaccinology pipeline for *S. aureus* vaccine development.^35^ The empirical arm uses transcriptomic, proteomic, and immunoproteomic analyses to identify infection-relevant antigens and evaluate their immunogenicity and protective potential in animal models. In parallel, the reverse-vaccinology arm employs in silico genome mining, epitope prediction, structural modeling, and molecular simulations to refine antigen–receptor interactions. Promising candidates from both arms are recombinantly expressed and tested *in vivo*, supporting the development of next-generation *S. aureus* vaccines.

In an effort to further decrease the number of microbial antigens considered, computer-aided selection tools based on available knowledge pertaining to successful vaccine antigens have been developed to extract common features, such as chemical properties of amino acid sequences^36^ or functional domains^37^ and apply this one step further to screen for vaccine candidates in microbial genome databases. The latter approach, protectome analysis, is the concept that protective vaccines should identify bacterial virulence factors that are harmful to the host and therefore share biological functions in addition to being immunogenic.^37^

Conversely, omics technologies are also enabling experimental approaches to vaccine development (Figure 1). They can be applied to preclinical models, but also of the populations directly concerned with the vaccine. As successful vaccination in mice has yet to translate to an effective human vaccine, this is a major benefit. As demonstrated above, the natural human immune response to *S. aureus* colonization and infection can be delineated with unprecedented detail and completeness.^38^ Antibody binding is a promising starting point for T-cell antigen selection, because in most instances, high affinity antibody development requires T-cell help.^39^ When focusing on *S. aureus* persisting inside host cells, detailed knowledge of both the transcriptional profile of the bacteria and proteome expressed by them is essential.^40^

**Reverse vaccinology** uses whole genome sequence data and bioinformatics algorithms to identify surface-exposed or secreted proteins likely targeted by the immune system.^28^ This method has been used with *S. aureus* to identify potential subunit vaccine candidates.^41^ In a pilot study, Glowalla et al. (2009) used used an easy reverse vaccinology method for *S. aureus* candidate epitopes inducing B- and T-cell mediated immunity identification.^42^ Instead of beginning with the whole bacterium genome, Glowalla et al. simply selected ten conserved surface exposed proteins for antigenicity evaluation and subsequently identified epitopes from fibronectin binding protein A (FnbpA), collagen adhesin (Cna), serine-rich adhesin for platelets (SraP) and elastin binding protein (EbpS) as likely targets but still required validation.^42^ Using the whole genome allows researchers to distinguish proteins important for bacterial survival or virulence without being poorly expressed *in vitro*.

**Genomic methodologies**, and especially pan-genomic reverse vaccinology, consist of comparing genome sequences of multiple isolates of the same pathogen species.^28^ This could be valuable to understand antigens that are broadly conserved across clinical strains, an important consideration when planning for broadly effective, universal vaccines.^5^ A detailed *in vivo* antigenic profiling of *S. aureus* has already identified over 60 antigenic proteins, primarily from proteins on the bacterial surface or those that are secreted.^43^ The capacity to computer screen thousands of proteins for properties such as surface exposure, conservation, and predicted immunogenicity prior to the costly step of *in vivo* studies greatly enhances the probability of success while also shortening overall timelines for development.

**Proteomic methodologies** have, for example, explored the use of techniques like trypsin shaving and subtractive proteome analysis (SUPRA) to characterize surface-exposed proteins and proteins that are recognized by opsonic antibodies.^44^ In one method, trypsin shaved bacterial cells are incubated in a hypotonic solution to reveal surface proteins that can be cleaved by trypsin and their resulting peptides analyzed for mass spectrometry.^44^ In SUPRA, protein extraction techniques are used that include immunoblotting with human sera (including depleted sera) to characterize proteins recognized by a specific antibody response. Proteomic methodologies have enabled the discovery of novel *S. aureus* antigens, such as AdcAau, and facilitate the differentiation between antigens that elicit protective antibody responses and those that do not, thereby providing crucial insights for the rational design of future multicomponent vaccines targeting diverse immune mechanisms.^45^

Immunoproteomics provides a wide-ranging view of the strength and dynamics of antibody binding to *S. aureus* proteins exposing their immunogenicity.^46^ Antibody profiling based on multiplex assays in patient cohorts is useful for testing and generating hypotheses.^47,48^ This has led to candidates to be considered for colloquial vaccination purposes. Antibody recognition of a small set of *S. aureus* antigens cosegregated with protection from sepsis in patients with *S. aureus* bacteremia.^47^

The integration of empirical and *in silico* methods allow us to maximize the extensive amount of data available and the diversity of the sources of that information in the vaccine discovery process. Genome-based computational methods, in combination with proteomics, allow us to identify critic proteins that are important in the host–pathogen relationship, even those that elicit weak immune responses or lack a well-defined structure. Additionally, although many intracellular bacterial proteins are not accessible to B cells, they may still serve as T-cell antigens. These should be prioritized and serve as a basis for the application of predictive tools to determine which targets are worth pursuing.

However, predictions generated by reverse vaccinology still require further experimental validation. In this context, ImunoProteomics of colonized or infected host was a more useful process of validating resulting bioinformatic predictions, rather than relying solely on low yield preclinical screens. This transition to omics driven computational approaches is a vast leap forward for vaccine discovery, allowing rational, rapid antigen selection and counteracting limitations such as the genetic diversity of *S. aureus* and complex antigenicity.

### 2.2 Immunoinformatic and Artificial Intelligence/Machine Learning in Epitope Prediction and Vaccine Design

Vaccine design is inherently complex, particularly when targeting pathogens with high genetic and antigenic variability. Traditionally, vaccine development has relied on empirical approaches, which are often costly, time-consuming, and inadequate for structurally diverse pathogens.^49^ These limitations are compounded by an incomplete understanding of pathogen–host interactions, the unavailability of permissive cell lines, and the lack of reliable animal models. Moreover, the development of vaccines against challenging diseases such as smallpox, HIV/AIDS, and tuberculosis continues to face significant scientific and logistical barriers.^50^

To overcome these challenges, a new generation of vaccine technologies has emerged. These include recombinant DNA technology, rational vaccinology, structural biology, conjugate vaccine platforms, and next-generation epitope-based designs. Vaccines produced through recombinant DNA methods are generally considered safe, effective, and more cost-efficient than traditional approaches, while also being well suited for the large-scale production of subunit vaccines.^51,52^ In parallel, the past two decades have seen a surge in computational tools that support immunotherapy and peptide-based drug discovery, accelerating the identification of novel targets for prophylactic and therapeutic intervention.^53^

The integration of genomic, structural, and computational biology has ushered in a new era of rational vaccine design. The combination of sequence-based analysis and recombinant DNA technology has facilitated the identification of protective B- and T-cell epitopes, enabling more precise and targeted vaccine strategies.^51,54^ Continued progress in this field requires multidisciplinary expertise and the coordinated application of bioinformatics, immunology, and molecular biology to develop next-generation vaccines capable of addressing global infectious disease threats.

Modern computational design acts as a driving force to enhance structural vaccinology, in which protein antigens are designed to construct new biomolecules with improved immunological properties.^55^ Ongoing advancements in vaccine development and diagnostics fuel the entire field of structural vaccinology (SV), reverse vaccinology (RV) and antigen recognition technology.^56^ Nevertheless, systems biology assists with what the host–pathogen interactions are and informs adjuvant capability, thus providing long-lasting immunity.^57^ In the positive light of these new technologies, rational vaccinology is a new and functionally relevant approach to create potent immunogens governing the induction of protective immunity, long-lasting immunity with this new technology, a synthetic peptide vaccine was developed for the treatment of asthma (Figures 2 and 3).^58^

**Figure 2:**
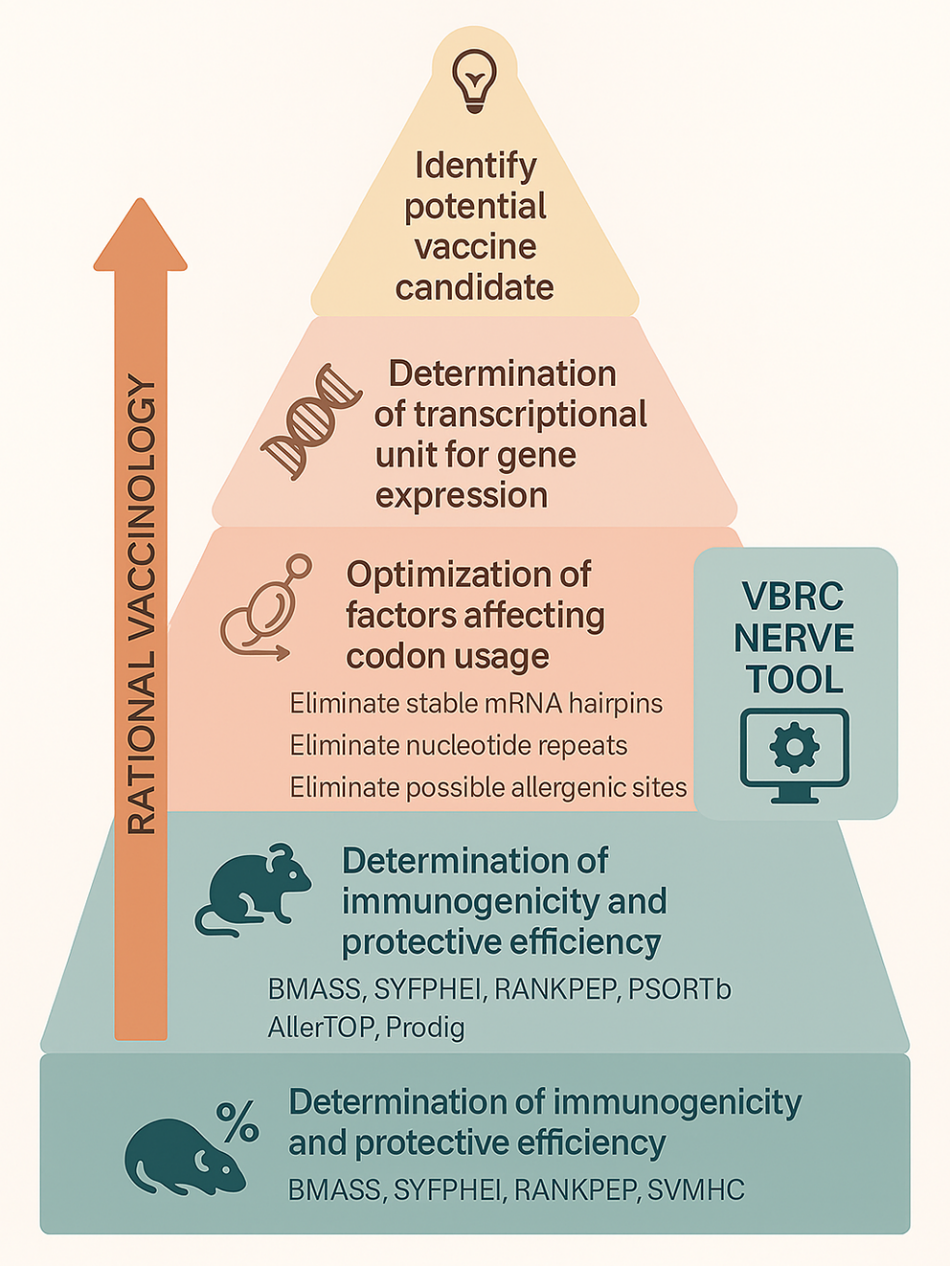
Rational vaccinology and its procedure to design vaccines through the VBRC NERVE tool. Illustration showing the steps involved in the identification of potential vaccine candidates through rational vaccinology. Each step uses different bioinformatics tools.

**Figure 3:**
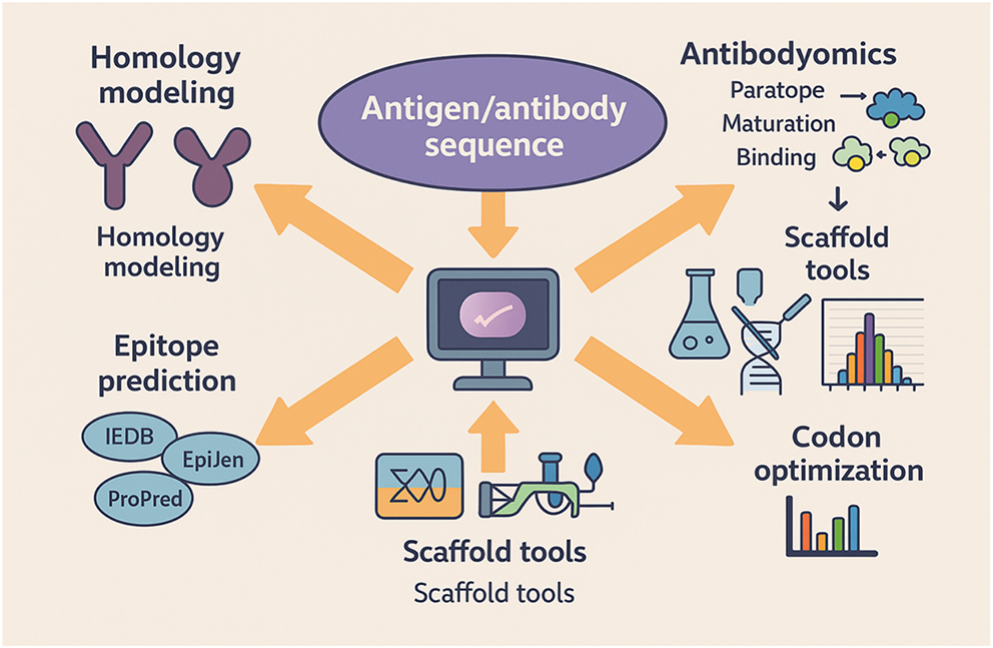
Computational strategies for the development of potent vaccine including antigen processing. Illustration showing the processing and screening of the antigen as an immunogen. The pathogen enters the cell and secretes proteins, which can be predicted by the Mature P software. Further processing of the protein is performed by proteosome and the antigenic peptide is released. This peptide, capable of binding MHC, can be predicted by MHCPred, RANKPEP, and SVMHC software. The peptide is then targeted as an epitope by the epitope prediction tools (IEDB, EpiJen, ProPred) and is selected as a potential vaccine candidate.

Immunoinformatic refers to the use of computational methods to predict B-cell and T-cell epitopes, study the binding affinity of B-cell or T-cell epitopes with human leukocyte antigen (HLA) alleles, and examine their overall antigenicity and immunogenicity.^59^ This technology is useful for designing multiepitope vaccines that can generate a potent and specific immune response while lowering the chance of undesirable or nonprotective responses.^60^ For example, one can use Tepitool and BepiPred programs to predict T-cell and B-cell epitopes, respectively, and then perform molecular docking studies to demonstrate binding properties with HLA molecules.^59,61^ While Martins et al. indicate that separate models for Gram-positive and Gram-negative bacteria are not required. PAPreC offers a comprehensive, adaptable, and robust framework to streamline and improve antigenicity prediction for diverse bacterial data sets.^62^

Artificial intelligence (AI) and machine learning (ML) represent a major advancement in vaccine development, offering powerful tools for analyzing large and complex data sets including antibody profiles, host genetics, and clinical outcomes to predict immune responses, optimize vaccine design, and identify the most immunogenic antigen candidates.^63^ For example, the AI-Immunology platform developed by Evaxion successfully identified novel *S. aureus* antigens that significantly reduced bacterial burden and induced robust protective antibody responses in large nonrodent animal models. These studies were conducted in naïve animals prior to *S. aureus* colonization, under conditions designed to simulate human infection.^64^

Recent studies have demonstrated that deep neural networks (DNNs), in combination with genomic and proteomic data, can predict B-cell epitope immunogenicity with up to 82% accuracy, as shown in work with SARS-CoV-2 surpassing traditional prediction methods. Similarly, ML models such as XGBoost have achieved accuracy rates as high as 97.6% in predicting T-cell epitope diversity, using physicochemical properties of peptide sequences as predictive features.^22,65^ These algorithms also leverage structural and genomic data to refine epitope selection and improve antigen stability, enhancing the likelihood of eliciting protective immunity.

Recent omics-guided and AI-enhanced pipelines have yielded a shortlist of *S. aureus* vaccine candidates with a markedly different antigenic profile from those that failed in past clinical trials. These include the integration of highly conserved antitoxins (such as Hla toxoids), surface lipoproteins with minimal sequence variability across clinical isolates (FhuD2, Csa1A), and immune-modulating secretion system effectors (EsxA/B), identified through interactome and *in vivo* expression profiling.^41^ AI-driven epitope ranking further prioritizes subdominant antigens less prone to immune imprinting and optimizes epitope diversity for broad HLA coverage. The resulting formulations, exemplified by the 4C-Staph platform expanded with network-RV-identified components, aim to elicit complementary protective mechanisms, neutralize key virulence determinants, and engage both humoral and Th1/Th17 cellular pathways.^66,67^ Such rationally assembled multicomponent constructs stand in contrast to earlier empirically selected candidates, offering a more robust strategy to circumvent historical pitfalls in *S. aureus* vaccinology.

Importantly, AI-driven antigen discovery offers new possibilities to overcome immune imprinting by selecting antigens that circumvent pre-existing, nonprotective immune memory. Additionally, AI can help design vaccine formulations with improved pharmacokinetic for instance, optimizing antigen release kinetics to support long-lasting immunity after a single dose.

### 2.3 Targeting Virulence Factors and Immune Modulators

A strategic shift in *S. aureus* vaccine development has redirected efforts from targeting only surface antigens an approach that has largely failed due to immune imprinting and bacterial evasion to focusing on the neutralization of secreted toxins.^10^ The bacterium expresses a variety of virulence determinants that contribute to pathogenesis, including surface-associated adhesins, capsular polysaccharides, exoenzymes, and exotoxins.^68,69^ Among these, secreted toxins play a central role by actively disrupting and evading host immune responses. Capsular polysaccharides from serotypes 5 and 8 (CP5 and CP8) have been explored as vaccine antigens when conjugated to the carrier protein (CP) CRM197. In murine models of bacteremia, lethal sepsis, and skin infection, immunization with CP5-CRM197 or CP8-CRM197 conjugates elicited robust antibody responses and provided protection against staphylococcal bacteremia.^68^ These findings suggest that the host immune system is capable of mounting an effective response against *S. aureus* when toxin-mediated immune evasion is neutralized, even in previously exposed individuals.

Key toxin targets include:

- **Alpha-Hemolysin (Hla):** Hla is a virulence factor associated with tissue damage and immune cell lysis.^70^ Vaccination with virus-like particles (VLPs) containing a linear neutralizing domain of Hla has been found to decrease lesion size and neutrophils infiltration in animals.^70^ Additionally, the use of combined Hla toxoids has conferred complete protection against lethal pneumonia in rabbits.^71,72^ Targeting Hla enables the neutralization of bacterial toxins, restores immune homeostasis, and facilitates an effective host immune response against infection.^11^
- **Leucocidins (e.g., Panton-Valentine Leucocidin (PVL), LukED, LukGH):** These toxins are well-known for their ability to lyse immune cells, primarily leukocytes, thereby impairing the host immune response and hindering the development of long-term immunity.^14,73,74^ Immunization with PVL components, for example, has been demonstrated to induce a strong antibody response, which contributes to protection against serious disease.^70,73^ The combination of Hla and leucocidin components in vaccine formulations has demonstrated potential to enhance immune responses against multiple toxins simultaneously.^74,75^

- **Staphylococcal Enterotoxin B (SEB):** SEB, recognized as a highly conserved superantigen and possible bioweapon, is an appealing target.^14^ Evidence shows mRNA-based vaccines and SEB-specific antibodies can provide in vivo potent, durable immune responses against the toxin, and help clear *S. aureus* from systemic infection in mice.^76^
- **Staphylococcal Protein A (SpA):** SpA serves as an important virulence factor that inhibits antibody and complement binding, disrupting functionality of B-cells.^14^ The mutation of SpA, and its application as a vaccine product (SpAmut/AS01), may unmask the pathogen to the immune system and therefore improve the immune response.^77^

This strategic ranking of toxins is specifically designed to address the challenge posed by *S. aureus* in disarming the host immune system. By neutralizing these important virulence factors, the intention is to re-enable the host’s protective functions. Beyond toxins, *S. aureus* expresses a variety of immune evasion proteins that interfere with neutrophil activity, inhibit complement deposition, and modulate B and T cell responses.^78^ Targeting immune evasion mechanisms, such as coagulase R domain (which assembles a fibrin shield for the bacterium), is a new approach.^79^ A monoclonal antibody developed against R domain was able to diminish the blood burden of bacteria in human volunteers, indicating this approach could be promising.^79^

Moreover, “subdominant antigens” are being investigated as a possible solution to immune imprinting. These antigens typically elicit a weaker initial immune response and may avoid triggering the same nonprotective recall responses associated with immunodominant antigens, thereby potentially circumventing defective immune memory.^80^ This is an important shift to eliciting antibodies that specifically neutralize the bacterium’s immune evasion strategies versus just targeting the presence of the bacterium.

Several antigens listed in Table 2, including alpha-hemolysin (Hla), leucocidins, staphylococcal protein A (SpA), and biofilm-associated proteins were originally developed and advanced toward clinical evaluation before the integration of multiomics and AI-based prioritization pipelines. Re-examination of these targets with genomics, transcriptomics, and proteomics has yielded important refinements in antigen selection and formulation design. For example, comprehensive pan-genomic analysis confirms that Hla is highly conserved among clinical isolates, while *in vivo* transcriptomic data sets and structural vaccinology approaches have mapped accessible neutralizing epitopes.^81^ Detoxified Hla toxoid (HlaH35L) is now a core component of the multivalent 4C-Staph formulation, where it is combined with adhesins and secretion-system effectors to elicit complementary immune mechanisms.^19^ Similarly, leucocidins such as LukED and PVL have been revisited through proteome-wide immune-profiling, enabling epitope selection that preserves immunogenicity while mitigating potential inflammatory toxicity.^82^

**Table 2:** Emerging Antigenic Targets for *S. aureus* Vaccines: Biological Rationale and Selection Criteria

**antigen and category**	**antigen**	**targeting**	**mechanism of action**	**citations**
**toxins**	α-hemolysin (Hla)	essential virulence factor, particularly in pneumonia; neutralizes bacterial toxins.	forms pores in host cells, causes cell death; vaccine aims to neutralize pore formation.	^11^
	leucocidins (PVL, LukED, LukGH)	kill immune cells (leukocytes), preventing effective immune responses and long-term immunity.	cause cell lysis by affecting membranal integrity of leukocytes.	^14^
	staphylococcal enterotoxin B (SEB)	highly conserved superantigen; potential bioweapon; critical for toxic shock syndrome.	potent superantigenic toxin, interacts with MHC II and TCR; vaccine aims to neutralize toxin.	^14^
**immune evasion proteins**	staphylococcal protein A (SpA)	undermines host immune responses; interferes with antibody/complement binding; causes B cell dysregulation.	binds to human VH3 antibodies, leading to B cell apoptosis or repertoire skewing; vaccine aims to ″unmask″ pathogen.	^14^
	coagulase R domain	forms protective fibrin shield, enabling evasion of phagocytic killing.	directs fibrinogen to bacterial surface, creating a fibrin shield.	^79^
**other key targets**	biofilm-associated proteins	crucial for *S. aureus* persistence, especially on medical devices; resists antimicrobials and host immunity.	promote biofilm formation, resist immune clearance; vaccine aims to prevent biofilm development.	^10^
	subdominant antigens	may bypass immune imprinting; elicit protective responses not subject to faulty recall.	elicit a weak initial immune response, potentially avoiding preferential recall of nonprotective antibodies.	^80^
	IsdA, IsdB, SdrD, SdrE (as part of multiantigen strategy)	surface antigens or secreted toxins detectable by immune response; antibodies can induce opsonophagocytosis.	induce opsonophagocytic antibodies; provide protection against invasive disease.	^1^

SpA, once considered a problematic antigen due to its Fc-binding capacity and B-cell superantigen activity, has been re-engineered into nontoxigenic variants that abolish Fc binding, unmasking the bacterium to effective opsonophagocytic killing.^83^ Biofilm-associated proteins (BAPs) are now prioritized using subtractive proteomic analysis of biofilm versus planktonic states, identifying surface-exposed, low-variability epitopes expressed during device-associated infections.^84^

Iron-regulated surface determinant proteins (IsdA, IsdB) have also been reassessed. While single-antigen approaches with IsdB failed clinically partly due to immune imprinting and poor antibody quality current multiantigen formulations combine IsdA/IsdB with antitoxins and Th1/Th17-promoting adjuvants to drive balanced cellular and humoral responses.^67,85^ AI-based epitope scoring supports such consensus cocktails, predicting improved breadth of protection and synergy between antigen classes. This combination of modern omics, structural modeling, and machine learning has transformed previously unsuccessful candidates into renewed, rationally optimized components of next-generation *S. aureus* vaccines.

### 2.4 Multivalent and Broad-Spectrum Vaccine Formulations

Given the repeated failure of single-antigen vaccines and the diverse array of *S. aureus* virulence factors, there is broad consensus that a multivalent vaccine targeting multiple antigens represents a more promising strategy.^19^ This approach resolves the issue of finding a single, well-conserved, and dominant antigen shared among all *S. aureus* strains that can be used as a target antigen for a universal vaccine.^10^ The future of *Staphylococcus* vaccines is likely to be multivalent vaccines with combinations of targets including toxins, T-cell epitopes and other antigens that can be expressed during different stages of the *S. aureus* life cycle and stages of infection.^11^

One area of growing interest involves targeting biofilm-associated proteins (BAPs). These proteins play critical roles in biofilm formation, maturation, and persistence, and are key contributors to the chronicity of infections particularly in the context of multidrug-resistant bacteria. Furthermore, targeting BAPs can also leverage drugs that could potentially lead to the discovery of new antibiofilm agents that improve therapy for persistent healthcare associated infection. BAPs are important in the nascent stability and durability of biofilms that protect the microbial community from both antibiotics and host immune response.^10,71^

Importantly, BAPs are directly linked to the pathogenicity of several bacteria, including *Acinetobacter baumannii*, a major cause of hospital-acquired infections. A hypothesis is that immunizing patients preoperatively with biofilm-upregulated antigens would encourage a shift in the host adaptive immune system toward an antibody-mediated response following infection with *S. aureus* in a biofilm mode of growth. This may be enough for the host to eliminate the infection in its early phase and bypass the damage of established biofilm communities.^86^ This approach recognizes *S. aureus*’s diverse infection modalities, aiming for holistic protection that covers both planktonic and biofilm forms of infection, a major clinical challenge that single-antigen, planktonic-focused vaccines have often overlooked.

Building on the lessons from past failures,^13,14,87^ next-generation *S. aureus* vaccine designs increasingly adopt multivalent strategies informed by omics data, structural vaccinology, and AI-based prediction.^22,28,41^ The 4C-Staph formulation, for example, integrates four antigens spanning toxins (Hla),^88^ conserved surface lipoproteins (FhuD2, Csa1A),^19^ and secretion-system effectors (EsxA/B),^19^ providing complementary protective mechanisms. Network-based reverse vaccinology has further expanded this antigen repertoire with novel interactome-filtered candidates, including subdominant antigens that are less likely to trigger nonprotective immune recall.^22,41,89^

These components are increasingly formulated with advanced adjuvants such as AS01, CpG-ODN, or other Th1/Th17-biasing system,^67,85,90^ which enhance opsonophagocytic activity, improve antibody functionality through Fc glycosylation modulation,^9,22^ and promote durable cellular immunity.^8,10^ This rational combination targets multiple disease stage from acute toxin-mediated pathology to biofilm-associated persistence^10,88^ and represents a decisive departure from narrowly focused, single-antigen approaches that have consistently failed in clinical trials.^10,13,14^

## 3 Integrated Vaccine Development Framework

The long-standing failure to develop an effective vaccine against *S. aureus* reflects a complex intersection of pathogen adaptability, immune evasion, and translational challenges. Despite extensive research, over 30 clinical trials, and numerous preclinical successes, no candidate has achieved durable protection in humans.^1,2^ This reality highlights that incremental improvements to traditional approaches are insufficient. A successful vaccine must address the bacterium multifaceted virulence, its ability to exploit immunological memory through immune imprinting, and the lack of robust correlates of protection in humans.

In this context, the integration of emerging scientific and technological paradigms offers a promising pathway forward. Novel strategies rooted in reverse vaccinology, systems biology, artificial intelligence, structural vaccinology, and precision immunology now enable a rational, data-driven approach to vaccine development. These advances shift the paradigm from empirical and reductionist models to one that is holistic, multidimensional, and anticipates the dynamic interplay between pathogen, host, and immune memory.^8,9^

### 3.1 Stepwise Integration of Technologies and Concepts

This section brings together the critical pillars explored throughout this review into a cohesive, translational pipeline. It begins with antigen discovery informed by omics and bioinformatics, progresses through computational epitope refinement and virulence targeting, and culminates in multivalent vaccine design optimized for human immunogenicity and clinical feasibility. Moreover, this framework embraces a precision-oriented mindset accounting for host factors such as age, HLA polymorphism, colonization status, and microbiome interactions thereby aligning with emerging principles of personalized vaccinology.^10^

Rather than relying on reductionist or single-target strategies, the integrated vaccinology approach leverages the synergistic potential of multicomponent design. This strategy aims to rationally develop vaccines that concurrently neutralize critical virulence determinants, circumvent immunological limitations such as nonprotective memory recall, and induce robust, long-lasting protective immunity across genetically and immunologically diverse human populations. The following sections delineate the core phases of this comprehensive framework, highlighting how advances in systems biology, structural vaccinology, and immune engineering can be translated into effective interventions against one of the most challenging pathogens in bacterial vaccine development.

#### 3.1.1 Reverse Vaccinology and Omics Discovery

The pipeline initiates with genome-wide and proteome-level screens employing pan-genomic comparison, transcriptomics during infection states (planktonic and biofilm), and surfome/secretome profiling. These unbiased analyses allow for the identification of conserved, surface-exposed, and immunologically relevant antigens, minimizing the selection of nonprotective targets influenced by immune imprinting.

#### 3.1.2 Immunoinformatic and AI-Assisted Epitope Prediction

Shortlisted antigens undergoes *in silico* refinement using B-cell and T-cell epitope prediction tools, enhanced by machine learning algorithms capable of predicting immunogenicity, population coverage (based on HLA diversity), and potential cross-reactivity. Artificial intelligence models aid in prioritizing subdominant or structurally novel epitopes with minimal pre-existing immune memory bias.

#### 3.1.3 Virulence Factor and Immune Modulation Targeting

The framework incorporates virulence-neutralization strategies by selecting toxins and immune evasion proteins as core antigens. These are prioritized for their role in acute disease pathogenesis and their ability to restore or preserve host immune function.

#### 3.1.4 Multivalent Antigen Formulation and Structural Vaccinology

The optimal vaccine formulation is multivalent, combining subdominant antigens, conserved surface proteins, and key toxins. Structural vaccinology and protein modeling are applied to design stabilized, highly immunogenic versions of selected antigens, engineered for proper folding, MHC binding, and enhanced B-cell receptor engagement.

#### 3.1.5 Adjuvant and Delivery Platform Selection

Given the need to redirect immune memory, the use of next-generation adjuvants that modulate Fc glycosylation and favor Th17 or cytotoxic responses is prioritized. mRNA, viral-like particles (VLPs), or nanoparticle-based delivery systems are considered to enhance antigen presentation and durability of response while allowing for rapid manufacturing scalability.

#### 3.1.6 Host-Microbiome and Personalized Profiling

Where feasible, human-derived omics data (e.g., from colonized vs uncolonized individuals, or resolved vs chronic infection) are integrated to refine candidate antigens and predict response profiles. This aligns with a precision vaccinology approach, considering microbiome composition, HLA typing, and immunosenescence in vulnerable populations (e.g., elderly, ICU patients).

For validation, the model emphasizes translational relevance by moving beyond traditional murine systems. Humanized mouse models and organoid platforms are utilized to better emulate human immune responses, while *ex vivo* assays employing neutrophils and PBMCs assess key functional outputs such as opsonophagocytic activity and T-cell activation. Multiplex serological profiling in clinical cohorts helps establish correlations between naturally acquired antibody repertoires and disease outcomes, and challenge studies in nonrodent models further substantiate immunogenicity and protective efficacy.

Altogether, this framework facilitates a coherent and rational progression from large-scale pathogen data to a refined, clinically viable multicomponent vaccine candidate. By integrating high-throughput discovery platforms, computational prediction, structural design, and systems immunology, this approach has the potential to overcome long-standing obstacles and enable the development of a robust and protective *S. aureus* vaccine one tailored not only to pathogen complexity, but also to the immunological diversity of the human population.

## 4 Conclusion and Perspectives

The enduring challenge of developing an effective vaccine against *S. aureus* underscores the intricate convergence of bacterial pathogenesis, immune evasion, and translational limitations that define this pathogen. Decades of intense research efforts have not yet yielded a licensed vaccine not due to isolated failures of individual candidates or platforms, but as a consequence of the need to fundamentally reconceptualize our approach to bacterial vaccinology. *S. aureus* is not a conventional pathogen; it is a highly adaptive commensal that has coevolved with the human immune system, exploiting mechanisms such as immune imprinting, antigenic redundancy, and phenotypic plasticity to persist in diverse host environments.

Recent advances in genomics, systems immunology, machine learning, and structural vaccinology provide unprecedented tools to address these complexities. The transition from empirically derived, single-antigen candidates to rationally designed, multicomponent formulations represents a pivotal evolution in the field. This shift must be coupled with a mechanistic understanding of host–pathogen interactions, predictive models of immunogenicity, and validation platforms that better reflect human immune physiology. Success in this domain will depend not only on technological innovation, but also on strategic integration across disciplines and institutions.

To that end, the development of a viable *S. aureus* vaccine will require sustained, collaborative efforts that bridge basic science, computational biology, clinical research, and regulatory expertise. Collaborative frameworks must span geographical and institutional boundaries, drawing on globally diverse data sets and human cohorts to ensure vaccine efficacy across heterogeneous populations. In the current landscape of escalating antimicrobial resistance and healthcare-associated infections, the urgency for preventive immunological interventions has never been greater.

Accelerating progress in this field will depend on coordinated investment in multiomics studies that elucidate the dynamics of colonization, infection, and immune evasion in human populations; the establishment of global antigen repositories and harmonized immunological assays to benchmark vaccine-induced responses; and the creation of translational pipelines that incorporate humanized preclinical models and systems-level correlates of protection. Moreover, the development and dissemination of open-access immunoinformatic platforms will be critical to enable epitope prediction, antigen selection, and adjuvant optimization tailored to high-priority bacterial pathogens. Equally essential is the design of consortia-led clinical trials capable of evaluating modular and multivalent vaccine candidates in stratified cohorts, thereby aligning with the principles of precision vaccinology.

Beyond synthesizing current evidence, this review integrates a critical appraisal of translational bottlenecks and formulates a data-driven framework for next-generation *S. aureus* vaccine design. By combining high-throughput omics, network-based reverse vaccinology, AI-assisted epitope prioritization, and structural vaccinology, we propose a stepwise strategy to overcome antigenic redundancy, immune imprinting, and virulence diversity. This perspective highlights concrete research priorities such as incorporating biofilm-associated antigens, targeting immune evasion proteins, and optimizing adjuvant selection to promote Th1/Th17 responses while emphasizing validation in humanized models. The field now approaches a pivotal juncture in which computational vaccinology, high-throughput antigen discovery, and human-centric immunological modeling are converging to transform the development of a safe and effective *S. aureus* vaccine from aspiration to tangible reality. Achieving this milestone will depend on coordinated, interdisciplinary alliances with true translational impact, ultimately addressing one of the most persistent gaps in infectious disease prevention and laying the foundation for vaccines against other immunologically complex bacterial pathogens in the postantibiotic era.

## References

[1] Clegg J., Soldaini E., McLoughlin R. M., Rittenhouse S., Bagnoli F., Phogat S. (2021). *Staphylococcus aureus* Vaccine Research and Development: The Past, Present and Future,
Including Novel Therapeutic Strategies. Front.
Immunol..

[2] Biolabs, C. *Staphylococcus aureus* Vaccines. n.d.. https://www.creative-biolabs.com/vaccine/staphylococcus-aureus-vaccines.htm.

[3] Mba I. E., Sharndama H. C., Anyaegbunam Z. K. G., Anekpo C. C., Amadi B. C., Morumda D., Doowuese Y., Ihezuo U. J., Chukwukelu J. U., Okeke O. P. (2023). Vaccine development for bacterial pathogens: Advances,
challenges and prospects. Trop. Med. Int. Health.

[4] Alkuraythi, D. Virulence Factors and Pathogenicity of *Staphylococcus aureus* . In Advances and Perspectives of Infections Caused by Staphylococcus aureus; Bustos-Martínez, J. ; Valdez-Alarcón, J. J. ; Hamdan-Partida, A. , Eds.; IntechOpen, 2024.

[5] Zhen X., Lundborg C. S., Zhang M., Sun X., Li Y., Hu X., Gu S., Gu Y., Wei J., Dong H. (2020). Clinical and
economic impact of methicillin-resistant *Staphylococcus
aureus*: a multicentre study in China. Sci. Rep..

[6] Thomsen, I. ; A Proctor, R. Chapter 56 - *Staphylococcus aureus* Vaccines. In Plotkin’s Vaccines (Eighth ed.); Orenstein, W. ; Offit, P. ; Edwards, K. M. ; Plotkin, S. , Eds.; Elsevier, 2023; pp 1087–1094.e1086.

[7] Proctor R. A. (2012). Challenges
for a Universal *Staphylococcus aureus* Vaccine. Clin. Infect. Dis..

[8] Hasanpour A.
H., Sepidarkish M., Mollalo A., Ardekani A., Almukhtar M., Mechaal A., Hosseini S. R., Bayani M., Javanian M., Rostami A. (2023). The global prevalence of methicillin-resistant *Staphylococcus aureus* colonization in residents of
elderly care centers: a systematic review and meta-analysis. Antimicrob. Resist. Infect. Control.

[9] Torres V. J. (2024). Interleukin
10 drives *Staphylococcus aureus* imprinting
and vaccine failure in murine models via antibody glycosylation. J. Clin. Invest..

[10] Tsai C. M., Caldera J., Hajam I. A., Liu G. Y. (2023). Toward an effective
Staphylococcus vaccine: why have candidates failed and what is the
next step?. Expert Rev. Vaccines.

[11] Analysis), I.-E. I. f. D. a. E. world needs a staph vaccine—new research could bring it a step closer. 2024. https://id-ea.org/the-world-needs-a-staph-vaccine-new-research-could-bring-it-a-step-closer/ (accessed June 16, 2025).

[12] Chand U., Priyambada P., Kushawaha P. K. (2023). *Staphylococcus aureus* vaccine strategy: Promise and challenges. Microbiol. Res..

[13] McNeely T. B., Shah N. A., Fridman A., Joshi A., Hartzel J. S., Keshari R. S., Lupu F., DiNubile M. J. (2014). Mortality
among
recipients of the Merck V710 *Staphylococcus aureus* vaccine after postoperative *S. aureus* infections: an analysis of possible contributing host factors. Hum. Vaccines Immunother..

[14] Scully I. L., Timofeyeva Y., Illenberger A., Lu P., Liberator P. A., Jansen K. U., Anderson A. S. (2021). Performance of a Four-Antigen *Staphylococcus aureus* Vaccine in Preclinical Models
of Invasive *S. aureus* Disease. Microorganisms.

[15] van
den Berg S., Bonarius H. P., van Kessel K. P., Elsinga G. S., Kooi N., Westra H., Bosma T., van der Kooi-Pol M. M., Koedijk D. G., Groen H., van Dijl J. M., Buist G., Bakker-Woudenberg I.
A. (2015). A human monoclonal antibody
targeting the conserved staphylococcal antigen IsaA protects mice
against *Staphylococcus aureus* bacteremia. Int. J. Med. Microbiol..

[16] Fattom A., Fuller S., Propst M., Winston S., Muenz L., He D., Naso R., Horwith G. (2004). Safety and immunogenicity of a booster
dose of *Staphylococcus aureus* types
5 and 8 capsular polysaccharide conjugate vaccine (StaphVAX) in hemodialysis
patients. Vaccine.

[17] Fowler V. G., Allen K. B., Moreira E. D., Moustafa M., Isgro F., Boucher H. W., Corey G. R., Carmeli Y., Betts R., Hartzel J. S., Chan I. S., McNeely T. B., Kartsonis N. A., Guris D., Onorato M. T., Smugar S. S., DiNubile M. J., Sobanjo-ter Meulen A. (2013). Effect of
an investigational vaccine for preventing *Staphylococcus
aureus* infections after cardiothoracic surgery: a
randomized trial. JAMA.

[18] Craven R. R., Gao X., Allen I. C., Gris D., Bubeck Wardenburg J., McElvania-Tekippe E., Ting J. P., Duncan J. A. (2009). *Staphylococcus
aureus* alpha-hemolysin activates the NLRP3-inflammasome
in human and mouse monocytic cells. PLoS One.

[19] Anderson A. S., Miller A. A., Donald R. G., Scully I. L., Nanra J. S., Cooper D., Jansen K. U. (2012). Development
of a multicomponent *Staphylococcus aureus* vaccine designed to counter
multiple bacterial virulence factors. Hum. Vaccines
Immunother..

[20] Matsunaga N. (2024). Evasion of
the Host Innate Immune System by Pathogenic Bacteria. Science.

[21] Fleischmann R. D., Adams M. D., White O., Clayton R. A., Kirkness E. F., Kerlavage A. R., Bult C. J., Tomb J. F., Dougherty B. A., Merrick J. M., McKenney K., Sutton G., Fitzhugh W., Fields C., Gocyne J. D., Scott J., Shirley R., Liu L.-I., Glodek A., Kelley J. M., Weidman J. F., Phillips C. A., Spriggs T., Hedblom E., Cotton M. D., Utterback T. R., Hanna M. C., Nguyen D. T., Saudek D. M., Brandon R. C., Fine L. D., Fritchman J. L., Fuhrmann J. L., Geoghagen N. S. M., Gnehm C. L., McDonald L. A., Small K. V., Fraser C. M., Smith H. O., Venter J. C. (1995). Whole-genome
random sequencing and assembly of *Haemophilus influenzae* Rd. Science.

[22] Bear A., Locke T., Rowland-Jones S., Pecetta S., Bagnoli F., Darton T. C. (2023). The immune evasion
roles of *Staphylococcus
aureus* protein A and impact on vaccine development. Front. Cell Infect. Microbiol..

[23] Chen Y., Liu Z., Lin Z., Lu M., Fu Y., Liu G., Yu B. (2023). The effect of *Staphylococcus aureus* on innate and adaptive immunity
and potential immunotherapy for *S. aureus*-induced osteomyelitis. Front. Immunol..

[24] Kane T. L., Carothers K. E., Lee S. W. (2018). Virulence Factor Targeting of the
Bacterial Pathogen *Staphylococcus aureus* for Vaccine and Therapeutics. Curr. Drug Targets.

[25] Pietrocola G., Nobile G., Rindi S., Speziale P. (2017). *Staphylococcus
aureus* Manipulates Innate Immunity through Own and
Host-Expressed Proteases. Front. Cell. Infect.
Microbiol..

[26] Karauzum H., Datta S. K. (2017). Adaptive Immunity Against *Staphylococcus
aureus*. Curr. Top. Microbiol.
Immunol..

[27] Wong
Fok Lung T., Chan L. C., Prince A., Yeaman M. R., Archer N. K., Aman M. J., Proctor R. A. (2022). *Staphylococcus
aureus* adaptive evolution: Recent insights on how
immune evasion, immunometabolic subversion and host genetics impact
vaccine development. Front. Cell. Infect. Microbiol..

[28] Bambini S., Rappuoli R. (2009). The use of genomics
in microbial vaccine development. Drug Discovery
Today.

[29] Lander E. S., Linton L. M., Birren B., Nusbaum C., Zody M. C., Baldwin J., Devon K., Dewar K., Doyle M., FitzHugh W., Funke R., Gage D., Harris K., Heaford A., Howland J., Kann L., Lehoczky J., LeVine R., McEwan P., McKernan K., Meldrim J., Mesirov J. P., Miranda C., Morris W., Naylor J., Raymond C., Rosetti M., Santos R., Sheridan A., Sougnez C., Stange-Thomann Y., Stojanovic N., Subramanian A., Wyman D., Rogers J., Sulston J., Ainscough R., Beck S., Bentley D., Burton J., Clee C., Carter N., Coulson A., Deadman R., Deloukas P., Dunham A., Dunham I., Durbin R., French L., Grafham D., Gregory S., Hubbard T., Humphray S., Hunt A., Jones M., Lloyd C., McMurray A., Matthews L., Mercer S., Milne S., Mullikin J. C., Mungall A., Plumb R., Ross M., Shownkeen R., Sims S., Waterston R. H., Wilson R. K., Hillier L. W., McPherson J. D., Marra M. A., Mardis E. R., Fulton L. A., Chinwalla A. T., Pepin K. H., Gish W. R., Chissoe S. L., Wendl M. C., Delehaunty K. D., Miner T. L., Delehaunty A., Kramer J. B., Cook L. L., Fulton R. S., Johnson D. L., Minx P. J., Clifton S. W., Hawkins T., Branscomb E., Predki P., Richardson P., Wenning S., Slezak T., Doggett N., Cheng J. F., Olsen A., Lucas S., Elkin C., Uberbacher E., Frazier M., Gibbs R. A., Muzny D. M., Scherer S. E., Bouck J. B., Sodergren E. J., Worley K. C., Rives C. M., Gorrell J. H., Metzker M. L., Naylor S. L., Kucherlapati R. S., Nelson D. L., Weinstock G. M., Sakaki Y., Fujiyama A., Hattori M., Yada T., Toyoda A., Itoh T., Kawagoe C., Watanabe H., Totoki Y., Taylor T., Weissenbach J., Heilig R., Saurin W., Artiguenave F., Brottier P., Bruls T., Pelletier E., Robert C., Wincker P., Smith D. R., Doucette-Stamm L., Rubenfield M., Weinstock K., Lee H. M., Dubois J., Rosenthal A., Platzer M., Nyakatura G., Taudien S., Rump A., Yang H., Yu J., Wang J., Huang G., Gu J., Hood L., Rowen L., Madan A., Qin S., Davis R. W., Federspiel N. A., Abola A. P., Proctor M. J., Myers R. M., Schmutz J., Dickson M., Grimwood J., Cox D. R., Olson M. V., Kaul R., Raymond C., Shimizu N., Kawasaki K., Minoshima S., Evans G. A., Athanasiou M., Schultz R., Roe B. A., Chen F., Pan H., Ramser J., Lehrach H., Reinhardt R., McCombie W. R., de la Bastide M., Dedhia N., Blocker H., Hornischer K., Nordsiek G., Agarwala R., Aravind L., Bailey J. A., Bateman A., Batzoglou S., Birney E., Bork P., Brown D. G., Burge C. B., Cerutti L., Chen H. C., Church D., Clamp M., Copley R. R., Doerks T., Eddy S. R., Eichler E. E., Furey T. S., Galagan J., Gilbert J. G., Harmon C., Hayashizaki Y., Haussler D., Hermjakob H., Hokamp K., Jang W., Johnson L. S., Jones T. A., Kasif S., Kaspryzk A., Kennedy S., Kent W. J., Kitts P., Koonin E. V., Korf I., Kulp D., Lancet D., Lowe T. M., McLysaght A., Mikkelsen T., Moran J. V., Mulder N., Pollara V. J., Ponting C. P., Schuler G., Schultz J., Slater G., Smit A. F., Stupka E., Szustakowki J., Thierry-Mieg D., Thierry-Mieg J., Wagner L., Wallis J., Wheeler R., Williams A., Wolf Y. I., Wolfe K. H., Yang S. P., Yeh R. F., Collins F., Guyer M. S., Peterson J., Felsenfeld A., Wetterstrand K. A., Patrinos A., Morgan M. J., de Jong P., Catanese J. J., Osoegawa K., Shizuya H., Choi S., Chen Y. J., Szustakowki J., International Human Genome Sequencing C. (2001). Initial sequencing
and analysis of the human genome. Nature.

[30] Bruno L., Cortese M., Rappuoli R., Merola M. (2015). Lessons from Reverse
Vaccinology for viral vaccine design. Curr.
Opin. Virol..

[31] Venter J. C., Adams M. D., Myers E. W., Li P. W., Mural R. J., Sutton G. G., Smith H. O., Yandell M., Evans C. A., Holt R. A., Gocayne J. D., Amanatides P., Ballew R. M., Huson D. H., Wortman J. R., Zhang Q., Kodira C. D., Zheng X. H., Chen L., Skupski M., Subramanian G., Thomas P. D., Zhang J., Gabor Miklos G. L., Nelson C., Broder S., Clark A. G., Nadeau J., McKusick V. A., Zinder N., Levine A. J., Roberts R. J., Simon M., Slayman C., Hunkapiller M., Bolanos R., Delcher A., Dew I., Fasulo D., Flanigan M., Florea L., Halpern A., Hannenhalli S., Kravitz S., Levy S., Mobarry C., Reinert K., Remington K., Abu-Threideh J., Beasley E., Biddick K., Bonazzi V., Brandon R., Cargill M., Chandramouliswaran I., Charlab R., Chaturvedi K., Deng Z., Di Francesco V., Dunn P., Eilbeck K., Evangelista C., Gabrielian A. E., Gan W., Ge W., Gong F., Gu Z., Guan P., Heiman T. J., Higgins M. E., Ji R. R., Ke Z., Ketchum K. A., Lai Z., Lei Y., Li Z., Li J., Liang Y., Lin X., Lu F., Merkulov G. V., Milshina N., Moore H. M., Naik A. K., Narayan V. A., Neelam B., Nusskern D., Rusch D. B., Salzberg S., Shao W., Shue B., Sun J., Wang Z., Wang A., Wang X., Wang J., Wei M., Wides R., Xiao C., Yan C., Yao A., Ye J., Zhan M., Zhang W., Zhang H., Zhao Q., Zheng L., Zhong F., Zhong W., Zhu S., Zhao S., Gilbert D., Baumhueter S., Spier G., Carter C., Cravchik A., Woodage T., Ali F., An H., Awe A., Baldwin D., Baden H., Barnstead M., Barrow I., Beeson K., Busam D., Carver A., Center A., Cheng M. L., Curry L., Danaher S., Davenport L., Desilets R., Dietz S., Dodson K., Doup L., Ferriera S., Garg N., Gluecksmann A., Hart B., Haynes J., Haynes C., Heiner C., Hladun S., Hostin D., Houck J., Howland T., Ibegwam C., Johnson J., Kalush F., Kline L., Koduru S., Love A., Mann F., May D., McCawley S., McIntosh T., McMullen I., Moy M., Moy L., Murphy B., Nelson K., Pfannkoch C., Pratts E., Puri V., Qureshi H., Reardon M., Rodriguez R., Rogers Y. H., Romblad D., Ruhfel B., Scott R., Sitter C., Smallwood M., Stewart E., Strong R., Suh E., Thomas R., Tint N. N., Tse S., Vech C., Wang G., Wetter J., Williams S., Williams M., Windsor S., Winn-Deen E., Wolfe K., Zaveri J., Zaveri K., Abril J. F., Guigo R., Campbell M. J., Sjolander K. V., Karlak B., Kejariwal A., Mi H., Lazareva B., Hatton T., Narechania A., Diemer K., Muruganujan A., Guo N., Sato S., Bafna V., Istrail S., Lippert R., Schwartz R., Walenz B., Yooseph S., Allen D., Basu A., Baxendale J., Blick L., Caminha M., Carnes-Stine J., Caulk P., Chiang Y. H., Coyne M., Dahlke C., Deslattes Mays A., Dombroski M., Donnelly M., Ely D., Esparham S., Fosler C., Gire H., Glanowski S., Glasser K., Glodek A., Gorokhov M., Graham K., Gropman B., Harris M., Heil J., Henderson S., Hoover J., Jennings D., Jordan C., Jordan J., Kasha J., Kagan L., Kraft C., Levitsky A., Lewis M., Liu X., Lopez J., Ma D., Majoros W., McDaniel J., Murphy S., Newman M., Nguyen T., Nguyen N., Nodell M., Pan S., Peck J., Peterson M., Rowe W., Sanders R., Scott J., Simpson M., Smith T., Sprague A., Stockwell T., Turner R., Venter E., Wang M., Wen M., Wu D., Wu M., Xia A., Zandieh A., Zhu X. (2001). The sequence
of the human genome. Science.

[32] Soltan M. A., Magdy D., Solyman S. M., Hanora A. (2020). Design of *Staphylococcus aureus* New Vaccine Candidates with
B and T Cell Epitope Mapping, Reverse Vaccinology, and Immunoinformatics. OMICS.

[33] Vita R., Overton J. A., Greenbaum J. A., Ponomarenko J., Clark J. D., Cantrell J. R., Wheeler D. K., Gabbard J. L., Hix D., Sette A., Peters B. (2015). The immune epitope database (IEDB)
3.0. Nucleic Acids Res..

[34] Doytchinova I. A., Flower D. R. (2007). Identifying candidate
subunit vaccines using an alignment-independent
method based on principal amino acid properties. Vaccine.

[35] Created by BioRender. Vieira Ferreira, Brenda. 2025 https://BioRender.com/wqccstn.

[36] Altindis E., Cozzi R., Di Palo B., Necchi F., Mishra R. P., Fontana M. R., Soriani M., Bagnoli F., Maione D., Grandi G., Liberatori S. (2015). Protectome
analysis: a new selective
bioinformatics tool for bacterial vaccine candidate discovery. Mol. Cell. Proteomics.

[37] Bröker B. M., van Belkum A. (2011). Immune proteomics
of *Staphylococcus
aureus*. Proteomics.

[38] Kolata J. B., Kühbandner I., Link C., Normann N., Vu C. H., Steil L., Weidenmaier C., Bröker B. M. (2015). The Fall
of a Dogma? Unexpected High T-Cell Memory Response to *Staphylococcus aureus* in Humans. J. Infect. Dis..

[39] Garzoni C., Francois P., Huyghe A., Couzinet S., Tapparel C., Charbonnier Y., Renzoni A., Lucchini S., Lew D. P., Vaudaux P., Kelley W. L., Schrenzel J. (2007). A global view
of *Staphylococcus aureus* whole genome
expression upon internalization in human epithelial cells. BMC Genomics.

[40] Oprea M., Antohe F. (2013). Reverse-vaccinology
strategy for designing T-cell epitope candidates for *Staphylococcus aureus* endocarditis vaccine. Biologicals.

[41] Salemi A., Pourseif M. M., Masoudi-Sobhanzadeh Y., Ansari R., Omidi Y. (2025). Proteome-wide
reverse vaccinology to identify potential vaccine candidates against *Staphylococcus aureus*. Mol.
Immunol..

[42] Glowalla E., Tosetti B., Krönke M., Krut O. (2009). Proteomics-Based Identification
of Anchorless Cell Wall Proteins as Vaccine Candidates against *Staphylococcus aureus*. Infect.
Immun..

[43] Etz H., Minh D. B., Henics T., Dryla A., Winkler B., Triska C., Boyd A. P., Sollner J., Schmidt W., von Ahsen U., Buschle M., Gill S. R., Kolonay J., Khalak H., Fraser C. M., von Gabain A., Nagy E., Meinke A. (2002). Identification of in vivo expressed
vaccine candidate antigens from *Staphylococcus aureus*. Proc. Natl. Acad. Sci. U.S.A..

[44] Sadones O., Kramarska E., Sainz-Mejias M., Berisio R., Huebner J., McClean S., Romero-Saavedra F. (2025). Identification of cross-reactive
vaccine antigen candidates in Gram-positive ESKAPE pathogens through
subtractive proteome analysis using opsonic sera. PLoS One.

[45] Jahantigh H. R., Faezi S., Habibi M., Mahdavi M., Stufano A., Lovreglio P., Ahmadi K. (2022). The Candidate Antigens to Achieving an Effective Vaccine
against *Staphylococcus aureus*. Vaccines.

[46] Stentzel S., Sundaramoorthy N., Michalik S., Nordengrün M., Schulz S., Kolata J., Kloppot P., Engelmann S., Steil L., Hecker M., Schmidt F., Völker U., Roghmann M.-C., Bröker B. M. (2015). Specific
serum IgG at diagnosis of *Staphylococcus aureus* bloodstream invasion is correlated with disease progression. J. Proteomics.

[47] Glasner C., van Timmeren M. M., Stobernack T., Omansen T. F., Raangs E. C., Rossen J. W., de Goffau M. C., Arends J. P., Kampinga G. A., Koedijk D. G., Neef J., Buist G., Tavakol M., van Wamel W. J., Rutgers A., Stegeman C. A., Kallenberg C. G., Heeringa P., van Dijl J. M. (2015). Low anti-staphylococcal IgG responses
in granulomatosis with polyangiitis patients despite long-term *Staphylococcus aureus* exposure. Sci. Rep..

[48] Sunita, Andaleeb S., Yogendra S., Shukla P. (2020). Computational
tools for modern vaccine development. Hum. Vaccines
Immunother..

[49] Arora G., Misra R., Sajid A. (2017). Model Systems
for Pulmonary Infectious Diseases: Paradigms of Anthrax and Tuberculosis. Curr. Top. Med. Chem..

[50] Kharkar, P. B. ; Talkar, S. S. ; Kadwadkar, N. A. ; Patravale, V. B. Chapter 11 - Nanosystems for oral delivery of immunomodulators. In Nanostructures for Oral Medicine; Andronescu, E. ; Grumezescu, A. M. , Eds.; Elsevier, 2017; pp 295–334.

[51] AlMalki F. (2024). In Silico Subtractive Proteome Analysis
to Design Multi-Epitope-Based Subunit Vaccine against Eikenella corrodens. J. Microbiol. Biotechnol..

[52] Hotez P. J., Molyneux D. H., Fenwick A., Ottesen E., Ehrlich
Sachs S., Sachs J. D. (2006). Incorporating a rapid-impact package
for neglected tropical diseases with programs for HIV/AIDS, tuberculosis,
and malaria. PLoS Med..

[53] Rappuoli R., Aderem A. (2011). A 2020 vision for vaccines
against HIV, tuberculosis
and malaria. Nature.

[54] Paladino A., Marchetti F., Rinaldi S., Colombo G. (2017). Protein design: from
computer models to artificial intelligence. WIREs Comput. Mol. Sci..

[55] Soria-Guerra R. E., Nieto-Gomez R., Govea-Alonso D. O., Rosales-Mendoza S. (2015). An overview
of bioinformatics tools for epitope prediction: implications on vaccine
development. J. Biomed. Inform..

[56] Sherman A. C., Mehta A., Dickert N. W., Anderson E. J., Rouphael N. (2019). The Future
of Flu: A Review of the Human Challenge Model and Systems Biology
for Advancement of Influenza Vaccinology. Front.
Cell. Infect. Microbiol..

[57] Bramwell V. W., Perrie Y. (2005). The rational design
of vaccines. Drug Discovery Today.

[58] Ranjbar K. J., Sarkoohi P., Shahbazi B., Babaei M., Ahmadi K. (2025). Bioinformatics
analysis of the in silico engineered protein vaccine with and without *Escherichia coli* heat labile enterotoxin adjuvant
on the model of Klebsiella pneumoniae. Sci.
Rep..

[59] Muhammad S. A., Guo J., Noor K., Mustafa A., Amjad A., Bai B. (2023). Pangenomic
and immunoinformatics based analysis of Nipah virus revealed CD4­(+)
and CD8­(+) T-Cell epitopes as potential vaccine candidates. Front. Pharmacol..

[60] Ullah N., Anwer F., Ishaq Z., Siddique A., Shah M. A., Rahman M., Rahman A., Mao X., Jiang T., Lee B. L., Bae T., Ali A. (2022). In silico
designed *Staphylococcus aureus* B-cell
multi-epitope vaccine
did not elicit antibodies against target antigens suggesting multi-domain
approach. J. Immunol. Methods.

[61] Shi, X. ; Tao, Y. ; Lin, S.-C. Deep Neural Network-Based Prediction of B-Cell Epitopes for SARS-CoV and SARS-CoV-2: Enhancing Vaccine Design through Machine Learning. arXiv:2412.00109. arXiv.org e-Print archive. https://arxiv.org/abs/2412.00109. 2024.

[62] Martins Y. C., Cerqueira E. C. M. O., Palumbo M. C., D F.
D. P., Custodio F. L., Trevizani R., Nicolas M. F. (2025). PAPreC: A Pipeline for Antigenicity
Prediction Comparison Methods across Bacteria. ACS Omega.

[63] Gude S., Abburi S. K., Gali P. K., Gorlagunta S. (2025). Advancing
single-shot vaccine design through AI and computational models. Transl. Regul. Sci..

[64] BioSpace . Evaxion and Undisclosed Collaborator Announce Encouraging Results for EVX-B1 Vaccine Antigens Against Staphylococcus aureus Infection. 2025. https://www.biospace.com/evaxion-and-undisclosed-collaborator-announce-encouraging-results-for-evx-b1-vaccine-antigens-against-staphylococcus-aureus-infection (accessed June 17, 2025).

[65] Nandi, S. Machine Learning in Immunology: From Epitope Prediction to Smarter Vaccine Design. AZoRobotics - Editorial Feature 2025. (acccessed June 17, 2025).

[66] Mancini F., Monaci E., Lofano G., Torre A., Bacconi M., Tavarini S., Sammicheli C., Arcidiacono L., Galletti B., Laera D., Pallaoro M., Tuscano G., Fontana M. R., Bensi G., Grandi G., Rossi-Paccani S., Nuti S., Rappuoli R., De Gregorio E., Bagnoli F., Soldaini E., Bertholet S. (2016). One Dose of *Staphylococcus aureus* 4C-Staph Vaccine Formulated
with a Novel TLR7-Dependent Adjuvant Rapidly Protects Mice through
Antibodies, Effector CD4+ T Cells, and IL-17A. PLoS One.

[67] Tsai C. M., Caldera J. R., Hajam I. A., Chiang A. W. T., Tsai C. H., Li H., Diez M. L., Gonzalez C., Trieu D., Martins G. A., Underhill D. M., Arditi M., Lewis N. E., Liu G. Y. (2022). Non-protective
immune imprint underlies failure of *Staphylococcus
aureus* IsdB vaccine. Cell Host
Microbe.

[68] Cheng B. L., Nielsen T. B., Pantapalangkoor P., Zhao F., Lee J. C., Montgomery C. P., Luna B., Spellberg B., Daum R. S. (2017). Evaluation of serotypes 5 and 8 capsular polysaccharides
in protection against *Staphylococcus aureus* in murine models of infection. Hum. Vaccines
Immunother..

[69] O’Riordan K., Lee J. C. (2004). *Staphylococcus aureus* capsular polysaccharides. Clin. Microbiol.
Rev..

[70] Joyner J. A., Daly S. M., Peabody J., Triplett K. D., Pokhrel S., Elmore B. O., Adebanjo D., Peabody D. S., Chackerian B., Hall P. R. (2020). Vaccination with VLPs Presenting
a Linear Neutralizing
Domain of *S. aureus* Hla Elicits Protective
Immunity. Toxins.

[71] Nor H., Mohd Z. (2024). Biofilm-associated proteins as potential drug targets. Afr. Res. J. Biosci..

[72] Wardenburg J. B., Schneewind O. (2008). Vaccine protection
against *Staphylococcus
aureus* pneumonia. J. Exp. Med..

[73] Brown E. L., Dumitrescu O., Thomas D., Badiou C., Koers E. M., Choudhury P., Vazquez V., Etienne J., Lina G., Vandenesch F., Bowden M. G. (2009). The Panton-Valentine leukocidin vaccine
protects mice against lung and skin infections caused by *Staphylococcus aureus* USA300. Clin. Microbiol. Infect..

[74] Hofstee M. I., Siverino C., Saito M., Meghwani H., Tapia-Dean J., Arveladze S., Hildebrand M., Rangel-Moreno J., Riool M., Zeiter S., Zaat S. A. J., Moriarty T. F., Muthukrishnan G. (2024). *Staphylococcus aureus* Panton-Valentine Leukocidin
worsens acute implant-associated osteomyelitis
in humanized BRGSF mice. JBMR Plus.

[75] Zhang Q., Jiang T., Mao X., Kim J. D., Ahn D. H., Jung Y., Bae T., Lee B. L. (2021). Development of Combination
Vaccine Conferring Optimal Protection against Six Pore-Forming Toxins
of *Staphylococcus aureus*. Infect. Immun..

[76] Luo F., Xu C., Zhang C., Tan A., Lu D., Luo P., Cheng P., Zhang W., Bai L., Yu C., Sun S., Zeng H., Zou Q. (2024). mRNA-based
platform for preventing
and treating *Staphylococcus aureus* by
targeted staphylococcal enterotoxin B. Front.
Immunol..

[77] Mandelli A. P., Magri G., Tortoli M., Torricelli S., Laera D., Bagnoli F., Finco O., Bensi G., Brazzoli M., Chiarot E. (2024). Vaccination with staphylococcal protein
A protects mice against systemic complications of skin infection recurrences. Front. Immunol..

[78] Askarian F., Wagner T., Johannessen M., Nizet V. (2018). *Staphylococcus
aureus* modulation of innate immune responses through
Toll-like (TLR), (NOD)-like (NLR) and C-type lectin (CLR) receptors. FEMS Microbiol Rev..

[79] Pozzi C., Bagnoli F., Rappuoli R. (2016). *Staphylococcus aureus* coagulase R domain, a new evasion
mechanism and vaccine target. J. Exp. Med..

[80] Grewal S., Hegde N., Yanow S. K. (2024). Integrating
machine learning to advance
epitope mapping. Front. Immunol..

[81] Bagnoli F., Fontana M. R., Soldaini E., Mishra R. P., Fiaschi L., Cartocci E., Nardi-Dei V., Ruggiero P., Nosari S., De Falco M. G., Lofano G., Marchi S., Galletti B., Mariotti P., Bacconi M., Torre A., Maccari S., Scarselli M., Rinaudo C. D., Inoshima N., Savino S., Mori E., Rossi-Paccani S., Baudner B., Pallaoro M., Swennen E., Petracca R., Brettoni C., Liberatori S., Norais N., Monaci E., Bubeck Wardenburg J., Schneewind O., O’Hagan D. T., Valiante N. M., Bensi G., Bertholet S., De Gregorio E., Rappuoli R., Grandi G. (2015). Vaccine composition
formulated with a novel TLR7-dependent adjuvant induces high and broad
protection against *Staphylococcus aureus*. Proc. Natl. Acad. Sci. U.S.A..

[82] Karauzum H., Haudenschild C. C., Moore I. N., Mahmoudieh M., Barber D. L., Datta S. K. (2017). Lethal CD4 T Cell Responses Induced
by Vaccination Against *Staphylococcus aureus* Bacteremia. J. Infect. Dis..

[83] Falugi F., Kim H. K., Missiakas D. M., Schneewind O. (2013). Role of protein
A in the evasion of host adaptive immune responses by *Staphylococcus aureus*. mBio.

[84] Di
Bonaventura G., Picciani C., Lupetti V., Pompilio A. (2023). Comparative
Proteomic Analysis of Protein Patterns of Stenotrophomonas maltophilia
in Biofilm and Planktonic Lifestyles. Microorganisms.

[85] Kim H. K., DeDent A., Cheng A. G., McAdow M., Bagnoli F., Missiakas D. M., Schneewind O. (2010). IsdA and IsdB antibodies protect
mice against *Staphylococcus aureus* abscess
formation and lethal challenge. Vaccine.

[86] Stranger-Jones Y. K., Bae T., Schneewind O. (2006). Vaccine assembly from surface proteins of *Staphylococcus aureus*. Proc.
Natl. Acad. Sci. U.S.A..

[87] Tsai C. M., Hajam I. A., Caldera J. R., Liu G. Y. (2022). Integrating complex
host-pathogen immune environments into *S. aureus* vaccine studies. Cell Chem. Biol..

[88] Wardenburg J. B., Schneewind O. (2008). Vaccine protection
against *Staphylococcus
aureus* pneumonia. J. Exp. Med..

[89] Proctor R. A. (2012). Challenges
for a universal *Staphylococcus aureus* vaccine. Clin. Infect. Dis..

[90] Buckley P.
T., Chan R., Fernandez J., Luo J., Lacey K. A., DuMont A. L., O’Malley A., Brezski R. J., Zheng S., Malia T., Whitaker B., Zwolak A., Payne A., Clark D., Sigg M., Lacy E. R., Kornilova A., Kwok D., McCarthy S., Wu B., Morrow B., Nemeth-Seay J., Petley T., Wu S., Strohl W. R., Lynch A. S., Torres V. J. (2023). Multivalent Human Antibody-Centyrin
Fusion Protein to Prevent and Treat Staphylococcus Aureus Infections. Cell Host Microbe.

